# Exacerbation of atherosclerosis by STX17 knockdown: Unravelling the role of autophagy and inflammation

**DOI:** 10.1111/jcmm.18402

**Published:** 2024-05-28

**Authors:** Xinyue Cui, Bo Wang, Dongjian Han, Mengdie Cheng, Peiyu Yuan, Pengchong Du, Yachen Hou, Chang Su, Junnan Tang, Jinying Zhang

**Affiliations:** ^1^ Department of Cardiology The First Affiliated Hospital of Zhengzhou University Zhengzhou Henan China; ^2^ Key Laboratory of Cardiac Injury and Repair of Henan Province Zhengzhou Henan China

**Keywords:** atherosclerosis, autophagy, inflammation, STING, syntaxin 17

## Abstract

Syntaxin 17 (STX17) has been identified as a crucial factor in mediating the fusion of autophagosomes and lysosomes. However, its specific involvement in the context of atherosclerosis (AS) remains unclear. This study sought to elucidate the role and mechanistic contributions of STX17 in the initiation and progression of AS. Utilizing both in vivo and in vitro AS model systems, we employed ApoE knockout (KO) mice subjected to a high‐fat diet and human umbilical vein endothelial cells (HUVECs) treated with oxidized low‐density lipoprotein (ox‐LDL) to assess STX17 expression. To investigate underlying mechanisms, we employed shRNA‐STX17 lentivirus to knock down STX17 expression, followed by evaluating autophagy and inflammation in HUVECs. In both in vivo and in vitro AS models, STX17 expression was significantly upregulated. Knockdown of STX17 exacerbated HUVEC damage, both with and without ox‐LDL treatment. Additionally, we observed that STX17 knockdown impaired autophagosome degradation, impeded autophagy flux and also resulted in the accumulation of dysfunctional lysosomes in HUVECs. Moreover, STX17 knockdown intensified the inflammatory response following ox‐LDL treatment in HUVECs. Further mechanistic exploration revealed an association between STX17 and STING; reducing STX17 expression increased STING levels. Further knockdown of STING enhanced autophagy flux. In summary, our findings suggest that STX17 knockdown worsens AS by impeding autophagy flux and amplifying the inflammatory response. Additionally, the interaction between STX17 and STING may play a crucial role in STX17‐mediated autophagy.

## 1 INTRODUCTION

Atherosclerosis (AS), a key manifestation of cardiovascular disease (CVD), is experiencing a surge in global incidence and mortality rates.^1^ AS is characterized by its chronic inflammation and abnormal lipids metabolism that affecting medium‐to‐large‐sized arteries^2^ and contributes significantly to coronary artery disease (CAD), cerebral infarctions and peripheral vascular disorders.^3^ Endothelial cells (ECs), vascular smooth muscle cells (VSMCs) and immune cells participate and affect the initiation and progression of AS.^4^ However, the dysfunction of ECs marks the initial stage of AS, where the accumulation of oxidized low‐density lipoprotein (ox‐LDL) exacerbates ECs damage and local inflammation.^5^ The current core strategy for AS treatment is to reduce circulating blood lipid levels, including low‐density lipoprotein cholesterol (LDL‐C) and non‐high density lipoprotein cholesterol (non‐HDL‐C).^6^ Lipid‐lowering medications include statins, PCSK9 inhibitors, ezetimibe and angiopoietin‐like 3 (ANGPTL3) inhibitors.^7^ Despite the use of potent lipid‐lowering agents, residual risk persists in AS patients. Therefore, a comprehensive understanding of AS mechanisms and the identification of potential therapeutic targets is crucial.

Autophagy, a vital process in eukaryotic cells, functions as a reparative mechanism.^8^ Autophagy is governed by autophagy‐related genes (Atg), which facilitates the formation and maturation of double‐membrane vesicles known as autophagosomes. Autophagosomes selectively engulf ageing cellular proteins and damaged organelles, subsequently fusing with lysosomes to promote the degradation of their contents.^9^ The term ‘autophagy flux’ encompasses the complete sequence of events, starting from autophagosome formation to final fusion with lysosomes.^10^ Extensive research has demonstrated the significant involvement of autophagy in numerous physiological and pathological processes, including cellular homeostasis, senescence, immune response, tumorigenesis, cardiovascular disorders and neurodegenerative conditions.^11^ However, the role of autophagy in AS remains contentious and is often underestimated. Autophagy exhibits dual effects on AS, which are contingent upon the level of autophagy activation.^12^ Moderate or controlled autophagy can prevent cellular damage, resulting in anti‐atherosclerotic effects. Nevertheless, the excessive autophagy activation may yield detrimental consequences, potentially leading to autophagic cell death.^13^ Growing evidence suggests that impeded autophagy flux plays a significant role in the progression of AS and the vulnerability of atherosclerotic plaques.^14^ Currently, the precise relationship between the dysfunction of ECs and the obstruction of autophagy flux in the development of AS remains uncertain.

Soluble N‐ethylmaleimide‐sensitive factor attachment protein receptors (SNAREs) constitute a group of highly conserved membrane‐related proteins crucial for specific membrane fusion processes.^15^ Syntaxin 17 (STX17), a member of the SNARE family, plays a pivotal role in mediating the fusion of autophagosomes and lysosomes.^16^ Research has shown that there is an elevated STX17 expression in cerebral ischemia/reperfusion neurons, while suppression of STX17 exacerbates neuronal damage via the autophagy‐lysosome pathway.^17^ Stimulator of interferon genes (STING) is a crucial signalling molecule in the innate immune response, facilitating immune defence mechanisms. The STING‐mediated inflammatory signalling pathway exhibits notable activation in various CVD, such as myocardial infarction, heart failure and myocarditis, indicating its involvement in CVD pathogenesis.^18^ Earlier investigations have proposed a potential interaction between STX17 and STING, which may participate in autophagy by STX17.^19^ However, the role of STX17 in inflammation and the interaction with STING in autophagy of ECs in the context of AS has not been reported yet.

Given the significance of autophagy and inflammation in AS progression, we postulated that STX17 may interact with STING to regulate autophagy and inflammation in ECs and affect AS. To validate this hypothesis, we utilized both in vivo and in vitro AS model systems, ApoE knockout (KO) mice fed a high‐fat diet and HUVECs treated with ox‐LDL, respectively, to explore the role and potential mechanism of STX17 and STING in AS.

## 2 MATERIAL AND METHODS

### 2.1 Animal experiment

Six‐week‐old SPF‐level male ApoE KO mice (*n* = 16) and 6‐week‐old C57BL/6J mice (*n* = 8) were selected for this study. The mice were divided into three groups: 6‐week ApoE KO mice were fed with western diet to constitute AS model group (G3, *n* = 8); 6‐week ApoE KO mice were fed with chow diet to constitute the control group (G2, *n* = 8); and 6‐week C57BL/6J mice were fed with chow diet to constitute the blank group (G1, *n* = 8).

The body weights of animals ranged from 28 to 34 g. Mice were raised in the Laboratory Animal Center of Gempharmatech Co., Ltd with animal licence number GPTAP20230317‐5. The rearing conditions included a temperature maintained between 22°C and 25°C, a relative humidity of 60%, and a light–dark cycle of 12 h. Throughout the treatment period, all mice had unrestricted access to both food and water. The mice were sacrificed after a 12‐week feeding period.

Plasma and aortic vessels were collected for subsequent analysis. Plasma samples were utilized to measure blood lipids using a blood biochemical analyser. Within each experimental group, aortic vessels from four mice were specifically designated for Oil Red O staining. Additionally, another set of aortic vessels from four mice underwent haematoxylin and eosin staining, Masson staining and Western blot analysis.

### 2.2 Haematoxylin and eosin staining

The aortic roots were embedded using an OCT embedding agent and rapidly frozen at −80°C. The frozen tissue was sliced into 10 μm sections using a frozen slicer. Subsequently, the sections were fixed, and the frozen slides were washed with OCT adhesive. The slides were then immersed in a haematoxylin staining solution for several minutes, followed by rinsing under running water to remove residual colour. To differentiate the cells, 5% acetic acid was applied, and the sections were subsequently stained with eosin. The stained slides were then dehydrated using a gradient ethanol series and sealed with neutral gelatin. Microscopic images were captured using a microscope system to facilitate further analysis.

### 2.3 Masson staining

The Masson staining procedure was conducted following the instructions provided in the kit (Modified Masson trichrome staining solution, Sbjbio, Nanjing, China). In summary, the slide designated for Masson staining was immersed in Bouin solution overnight at room temperature and subsequently rinsed with running water until the yellow colour disappeared. The slide was then treated with an azure blue staining solution for 2–3 min, followed by thorough washing with water. Mayer's haematoxylin staining solution was applied for 2–3 min, and after staining, the slide was washed. Differentiation was achieved by treating the slide with acidic ethanol for a few seconds, followed by a 10‐min rinse with running water. Next, the slide underwent staining with magenta staining solution for 10 min, followed by rinsing with distilled water. Treatment with phosphomolybdic acid solution for 10 min preceded the direct application of aniline blue staining solution for 5 min. A brief 2‐min treatment with weak acid solution followed. Finally, the slide was dehydrated using a gradient ethanol series and sealed with neutral gelatin. Microscopic images were captured using the microscope system for subsequent analysis.

### 2.4 Oil red O staining

The aortic vessels were immersed in 60% isopropanol for several minutes and then stained with Oil Red O solution at 37°C for 5 min. Subsequently, the aortic vessels underwent destaining using 60% isopropanol for several minutes. Microscopic images were captured using the microscope system for further analysis.

### 2.5 Cell culture

HUVECs sourced from MeisenCTCC (Zhejiang, China) were cultured in an endothelial cell medium (ECM; ScienCell, USA). The culture medium was supplemented with 5% FBS (ScienCell), 1% endothelial cell growth supplement (ScienCell) and 1% penicillin and streptomycin (ScienCell). HUVECs were maintained in a humidified incubator at 37°C with 5% CO_2_. Cells at passages 2–7 were utilized for subsequent investigations. To model AS in vitro, HUVECs were treated with oxidized low‐density lipoprotein (YB‐002, Yiyuan Biotechnologies, Guangzhou, China).

### 2.6 Lentivirus infection

Lentiviruses‐shSTX17 (pHBLV‐U6‐MCS‐CMV‐ZsGreen‐PGK‐PURO, shSTX17 sequence: 5′‐CATGACTGTTGGTGGAGCATTTCAT‐3′, 1.5 × 10^8^ TU/mL) were custom‐designed by HANBIO (Shanghai, China). At passage 2 of HUVECs, lentivirus transfection was conducted. HUVECs were seeded in a 6‐well plate in advance (8–10 × 10^4^ cells/well) with a cell density of 30%–50%. The medium was then switched to ECM containing polybrene and the virus solution (MOI = 10–30) for 24 h. Following transfection for 48 h, fresh ECM‐containing puromycin (2 μg/mL) was applied for an additional 24–48 h to select positively transfected cells. RNA or cell protein was subsequently extracted to measure STX17 mRNA or protein content in HUVECs. It is worth noting that for HUVECs transfected with mRFP‐GFP‐LC3 adenovirus, lentiviruses‐shSTX17 without GFP were utilized in the transfection process.

### 2.7 Autophagy flux analysis

HUVECs previously infected with shRNA‐STX17, shRNA‐NC lentivirus or the control, cultured in confocal dishes, were further transfected with mRFP‐GFP‐LC3 adenovirus (HANBIO, Shanghai, China) at an MOI of 500. Following a 24‐h transfection period, the cells were subjected to a 48‐h culture with ox‐LDL. Subsequently, the cells were fixed using 4% paraformaldehyde and examined using a confocal microscope (LSM 980, Zeiss, Germany). The quantification of mRFP and GFP puncta was performed based on analysis of four different images within each experimental group.

### 2.8 Treatment of HUVECs with small interfering RNA (siRNA)

SiRNA oligonucleotides specific for human STING were procured from HANBIO (Shanghai, China). HUVECs were transfected with STING‐siRNA or scrambled siRNA using Lipofectamine^TM^ RNAiMAX (Thermo Scientific, USA) following the manufacturer's instructions. Briefly, HUVECs were cultured until they reached 60% confluence. Transfection was carried out with 50 nM siRNAs for 6 h, after which the cells were switched to fresh ECM. Subsequently, RNA and protein extraction procedures were conducted on the transfected cells.

### 2.9 Real‐time PCR analysis

Total RNA was extracted from HUVECs using TRIzol reagent (Invitrogen) following the manufacturer's instructions. For mRNA detection, cDNA was synthesized using 1 μg of total RNA with HiScript III All‐in‐one RT SuperMix Perfect for qPCR (Vazyme, Nanjing, China). Subsequently, quantitative reverse transcription‐polymerase chain reaction (qRT‐PCR) was performed with ChamQ Universal SYBR qPCR Master Mix (Vazyme, Nanjing, China). The relative standard curve method (2^−ΔΔCT^) was employed to determine the relative mRNA expression, using *GAPDH* as the reference gene. The primer sequences used were as follows: STX17: forward, 5′‐CTCCGATCCAATATCCGAGAAA‐3′ and reverse, 5′‐GAGGCTGTAGCAAAGTTTCTTC‐3′; and GAPDH: forward, 5′‐TGACATCAAGAAGGTGGTGAAGCAG‐3′ and reverse, 5′‐GTGTCGCTGTTGAAGTCAGAGGAG‐3′; and STING: forward, 5′‐GCCCTGTTGCTGCTGTCCATC‐3′ and reverse, 5′‐GGATGTTCAGTGCCTGCGAGAG‐3′.

### 2.10 Western blot

For protein extraction from HUVECs, cells were collected, and 100 μL of lysis buffer per 10^6^ cells was added to the EP tube. After mixing and incubating on ice for 30 min, the samples were centrifuged at 12,000 rpm for 10 min, and the supernatant was collected. Protein concentration was measured using a BCA kit (PC0020, Solarbio, Beijing, China). To the samples, 5× protein loading buffer (LT101, Yamei, Shanghai, China) was added, followed by boiling for 10 min. SDS‐PAGE gels with different concentrations were prepared based on the molecular weight of the target proteins, and the sample volume was adjusted according to the measured protein concentration. The samples were loaded into the sample wells of SDS‐PAGE gels for electrophoresis. The separated proteins were then transferred to PVDF membranes (IPVH00010, Millipore, USA). Subsequently, the membranes were blocked in 5% skim milk (1172GR100, BioFroxx, Germany) for 90–120 min. After washing the membranes three times with TBST, they were incubated overnight at 4°C with different antibodies, including those against STX17 (1:500, Sigma, HPA001204), LC3 I/II (1:1000, CST, 12741), SQSTM1/p62 (1:10000, Abcam, ab109012), LAMP1 (1:1000, Abcam, ab108597), Cathepsin D (1:1000, Abcam, ab6313), STING (1:1000, Abcam, ab239074), β‐tubulin (1:20000, Proteintech, 66,240‐1‐Ig), GAPDH (1:1000, ZSGB‐BIO, TA‐08) and β‐actin (1:1000, ZSGB‐BIO, TA‐09). After another three washes with TBST, the membranes were incubated for 1 h at room temperature with diluted HRP—goat anti‐rabbit IgG or HRP—goat anti‐mouse IgG. Following three additional washes, the membranes were treated with an ECL luminescent working solution. The relative expression levels of the target proteins were determined using ImageJ software.

### 2.11 Immunoprecipitation analysis

HUVECs were cultured in a 10 cm culture dish. The cells were washed three times with PBS, and cell lysis buffer for immunoprecipitation (IP) (Beyotime, Shanghai, China) was added for 30 min. Subsequently, the mixture was centrifuged at 12,000 rpm for 10 min to obtain the supernatant. Pierce^TM^ Protein A/G Magnetic Beads (Thermo Scientific, USA) were utilized following the provided instructions. Briefly, the beads were mixed for 1 min, and each group took 50 μL of beads. The beads were washed three times with wash buffer and then incubated with the corresponding antibody at room temperature for 30 min. After three additional washes, the beads were incubated with the protein solution overnight at 4°C. On the second day, the beads were washed 3–5 times, and 1× loading buffer was added at 100°C for 10 min. Subsequently, the beads were discarded, and the supernatant was collected for Western blot analysis.

### 2.12 Cell growth assay

The cell growth curve was established using the Cell Counting Kit‐8 (CCK8) assay (DojinDo, Japan). HUVECs were plated in 96‐well plates at a density of 2000 cells per well. The cells were treated with 10 μL of CCK8 solution for 4 h simultaneously over the course of 6 days. Subsequently, the absorbance at a wavelength of 450 nm was analysed.

### 2.13 HUVECs scratch wound assay

The migration of HUVECs was assessed through a scratch wound healing assay. HUVECs were seeded in 6‐well plates at a density of 10 × 10^4^ cells per well and treated with or without ox‐LDL for 48 h. A scratch was generated in the confluent cell layer using a 200 μL pipette tip. Following two washes with PBS to eliminate loose cells, HUVECs were cultured in fresh ECM containing 3% FBS and maintained at 37°C. Images were captured at 6 and 12 h, and the measurements were performed using ImageJ software.

### 2.14 ECs tube‐formation assay

Matrigel Matrix (Corning, NY, USA) was added to a 96‐well plate and incubated at 37°C for 30 min to gel. Then, with or without ox‐LDL treated HUVECs (8000 cells/well) were added to the 96‐well plate (five replicates per group). After incubation at 37°C for 4 h, tube formation was observed using an inverted microscope. The total branch length and number of nodes were measured by ImageJ software.

### 2.15 ELISA of HUVECs supernatants

The expression of proinflammatory cytokines (TNF‐α, IL‐6, IL‐8 and MCP‐1) was quantified in the supernatant of ox‐LDL‐induced HUVECs using an ELISA kit obtained from Elabscience (Wuhan, China), following the manufacturer's instructions. The optical density was measured using a microplate reader at 450 nm.

### 2.16 Immunofluorescence staining

HUVECs were inoculated into a 2‐well plate specifically designed for laser confocal microscopy (ibidi, Germany). The staining products employed were sourced from Beyotime. The cells were fixed with Immunol Staining Fix Solution and subsequently washed three times with Immunol Staining Wash Buffer. Next, Immunostaining Permeabilization Solution with Triton X‐100 was applied for 5 min, followed by an additional three washes. Subsequently, QuickBlock™ Blocking Buffer for Immunol Staining was utilized for a duration exceeding 15 min, with 1–2 subsequent washes. The cells were then incubated overnight with antibodies against STX17 and STING. Following washes, the cells were incubated with goat anti‐mouse IgG H&L (Alexa Fluor® 594) and goat anti‐rabbit IgG H&L (Alexa Fluor® 488) for 1 h at room temperature. Afterward, the cells were washed three times with a wash buffer. Finally, several drops of Antifade Mounting Medium with DAPI were added to stain the nucleus. Imaging of HUVECs was conducted using a confocal microscope (LSM 980, Zeiss, Germany).

### 2.17 Statistical analysis

All data were presented as mean ± standard, and statistically analysed using SPSS 21.0 software. *T*‐test was used for comparison between two groups, while one‐way analysis of variance (ANOVA) was used for comparison between three or more groups. *p* < 0.05 was considered statistically significant.

## 3 RESULTS

### 3.1 Constructs in vivo AS model and STX17 expression is upregulated in AS model

To ascertain the involvement of STX17 in the progression of AS, we investigated the changes in STX17 expression in an in vivo AS model. The AS model group (G3) was established by feeding 6‐week ApoE KO mice a high‐fat and high‐cholesterol diet for 12 weeks. Concurrently, control groups were formed, including 6‐week C57BL/6J mice (G1) and ApoE KO mice (G2) fed a normal chow diet for 12 weeks.

**FIGURE 1:**
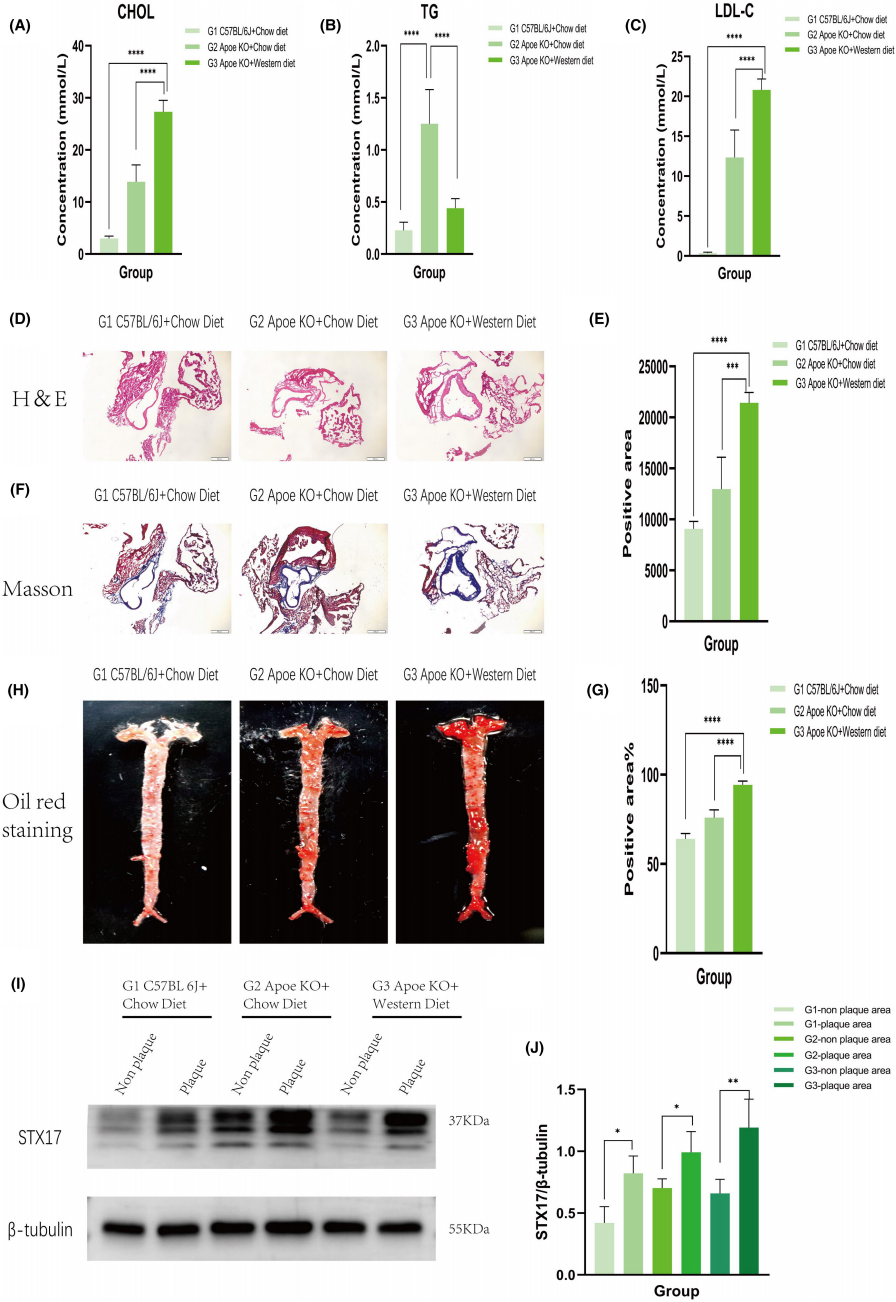
In vivo AS model construction and increased expression level of STX17 in AS model. Six‐week‐old ApoE KO mice were fed a high‐fat diet to induce AS in the AS model group (G3, *n* = 4), while 6‐week‐old C57BL/6J mice (G1, *n* = 4) and ApoE KO mice (G2, *n* = 4) were fed a normal chow diet for 12 weeks as control groups. (A–C) Plasma lipid levels, including CHOL (A), TG (B) and LDL‐C (C), were measured using a blood biochemical analyser at 18 weeks. (D–H) Aortic vessels were extracted for haematoxylin and eosin staining (D and E), Masson staining (F and G), and oil red O staining (H). Representative images of staining are shown, and the scale bar represents 500 μm. (I and J) The expression level of STX17 in aortic vessels was assessed by Western blots and is presented in a bar graph (*n* = 4). Data are represented as mean ± SD. *p < 0.05, **p < 0.01, ***p < 0.001, ****p < 0.0001 versus control groups.

Firstly, blood lipid values were measured and analysed in the three groups. The results demonstrated a significant increase in plasma cholesterol (CHOL) levels (Figure 1A) and LDL‐C levels (Figure 1C) in the G3 group compared to the G1 and G2 groups (both *p* < 0.001), while triglyceride (TG) levels (Figure 1B) were significantly reduced in the G3 group (*p* < 0.001). This reduction could be attributed to the accumulation of TG in the liver caused by a high‐fat diet. Histopathological analyses, including haematoxylin and eosin staining (Figure 1D,E) and Masson staining (Figure 1F, G) of aortic vessels, indicated no significant pathological changes in the G1 group, mild plaques in the G2 group and significant plaques in the G3 group (both *p* < 0.05). Oil Red O staining results (Figure 1H) revealed multiple pronounced plaques in the G3 group compared to the G1 group, which exhibited no significant pathological changes. These findings collectively validated the successful construction of the in vivo AS model in the G3 group.

We then assessed STX17 expression in both plaque and non‐plaque areas of aortic vessels. The results demonstrated significantly elevated expression of STX17 in the plaque area compared to the non‐plaque area, with the most substantial increment observed in the plaque area of the G3 group (Figure 1I,J).

### 3.2 Constructs in vitro AS model and STX17 expression is upregulated in AS model

**FIGURE 2:**
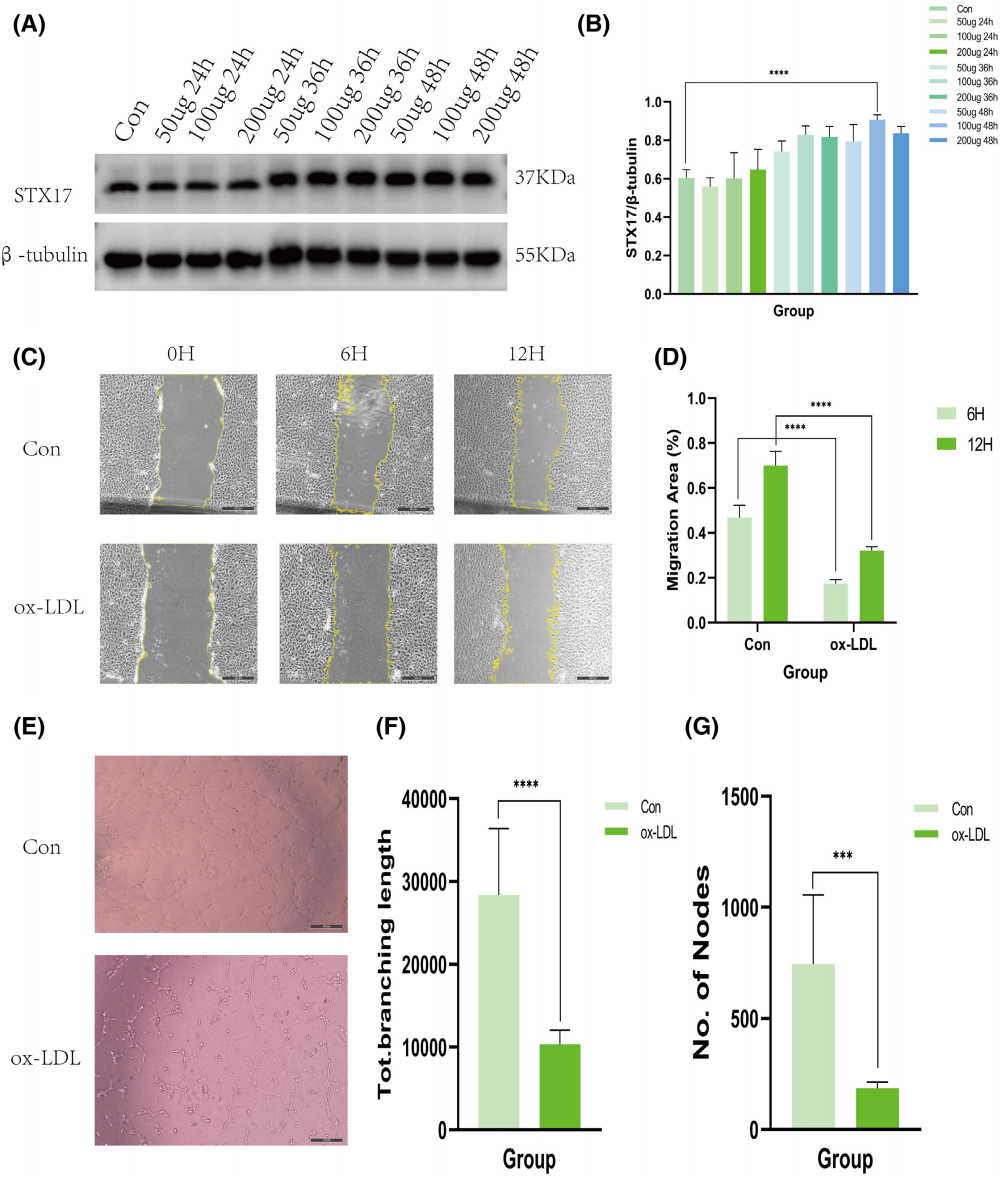
In vitro AS model construction and elevated expression of STX17. (A and B) HUVECs were treated with ox‐LDL at different concentrations (50 μg/mL, 100 μg/mL and 200 μg/mL) and for varying durations (24 h, 36 h and 48 h). The expression level of STX17 in aortic vessels was assessed using Western blots and presented in a bar graph (*n* = 3). (C, D) Scratch experiments were conducted in HUVECs with or without ox‐LDL treatment (100 μg/mL for 48 h). Representative micrographs are displayed and analysed in a bar graph (*n* = 6). (E–G) The tube formation ability of HUVECs with or without ox‐LDL treatment (100 μg/mL for 48 h) was evaluated. Representative micrographs are presented and analysed in a bar graph (*n* = 6). Data are represented as mean ± SD. ***p < 0.001, ****p < 0.0001 versus control.

To further investigate the role of STX17 in AS, we utilized ox‐LDL‐stimulated HUVECs to establish an in vitro AS model. Endothelial cell injury and endothelial dysfunction is believed to be the initiation of AS.^20^ Ox‐LDL stimulate HUVECs is the most commonly used method to construct ECs injury and dysfunction, which is the in vitro AS model.^21^ Various concentrations of ox‐LDL (50 μg/mL, 100 μg/mL and 200 μg/mL) were applied for different durations (24 h, 36 h and 48 h) based on previous studies. The results demonstrated a consistent increase in the expression level of STX17 across all groups after ox‐LDL treatment, with the most significant augmentation observed following 100 μg/mL ox‐LDL treatment for 48 h (Figure 2A,B). This concentration and treatment time were chosen for subsequent in vitro studies.

We treated HUVECs with ox‐LDL to simulate ECs injury or dysfunction in vitro, scratch and tube formation experiments were conducted to assess migration and angiogenesis ability of ECs.^22^ Scratch experiment is used to detect cell migration ability, while tube formation experiment is used to detect angiogenesis ability.^23^ Scratch experiments revealed a significant inhibition of scratch closure at 6 and 12 h after ox‐LDL treatment compared to the control group (Figure 2C,D). Concurrently, tube formation experiments (Figure 2E–G) demonstrated that ox‐LDL treatment attenuated tube formation in HUVECs. Both sets of experiments confirmed the successful development of the in vitro AS model.

The aforementioned results collectively suggest the involvement of STX17 in AS. Subsequently, we aimed to elucidate the specific role of STX17 in the initiation and progression of AS.

### 3.3 Knockdown of STX17 impairs HUVECs migration and angiogenesis

**FIGURE 3:**
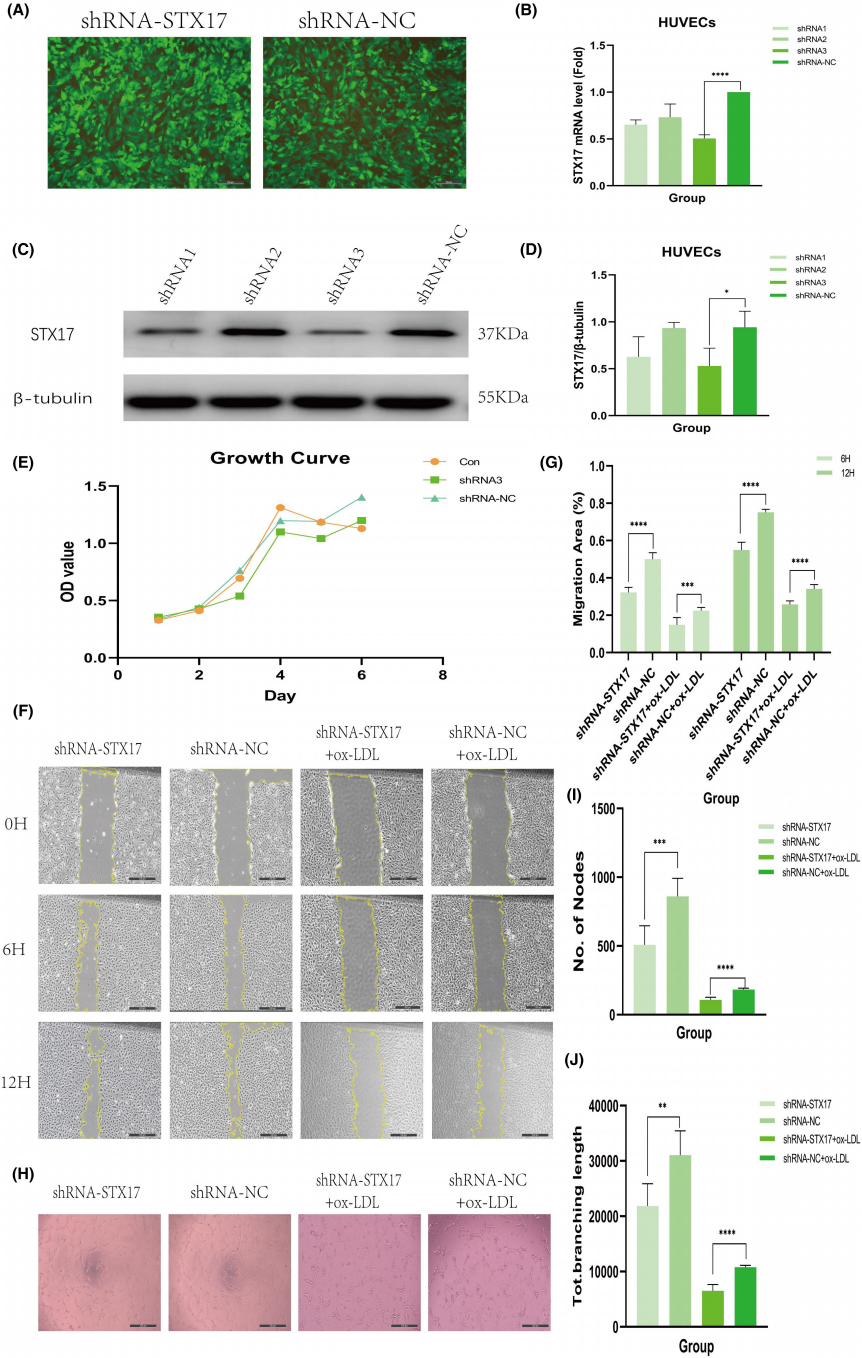
Knockdown of STX17 exacerbates HUVECs migration and angiogenesis. At passage 2 of HUVECs, cells were infected with shRNA‐STX17 or shRNA‐NC lentivirus, respectively. (A) Representative fluorescent micrographs are shown. Scale bar = 50 μm. Green: GFP. (B–D) The efficiency of STX17 knockdown was evaluated with PCR (B) and Western blot (C and D). STX17 expression levels are shown in Western blots (C) and presented in a bar graph (D, *n* = 3). (E) Growth curves of the three groups of HUVECs were performed using CCK8 methods for 6 days. (F–G) Scratch experiments were conducted in transfected HUVECs with or without ox‐LDL treatment (100 μg/mL for 48 h). Representative micrographs are displayed and analysed in a bar graph (n = 6). (H–J) Tube formation ability of transfected HUVECs with or without ox‐LDL treatment (100 μg/mL for 48 h) was evaluated. Representative micrographs are shown and analysed in a bar graph (*n* = 6). Microscope magnification: 20×. Data are represented as mean ± SD. *p < 0.05, **p < 0.01, ***p < 0.001, ****p < 0.0001 versus shRNA‐NC.

To explore the impact of STX17 on HUVECs in the context of AS, we transfected primary HUVECs with shRNA‐STX17 lentivirus to knock down STX17 expression. Following transfection with either shRNA‐STX17 or shRNA‐NC lentivirus, approximately 90% of HUVECs exhibited GFP fluorescence (Figure 3A), indicating successful transfection. Subsequently, transfection efficiency was further validated through PCR and WB. The results revealed that the expression level of STX17 in HUVECs transfected with shRNA‐STX17 was approximately 50% of that in HUVECs transfected with shRNA‐NC lentivirus (Figure 3B–D).

We investigated the role of STX17 in the viability of HUVECs by analysing cell activity and damage in STX17‐knockdown HUVECs subjected to ox‐LDL treatment. Initially, we assessed the growth trend of HUVECs after STX17 knockdown using the CCK8 method. The findings indicated no significant difference in the growth trend among cell groups, suggesting that cell transfection did not impact migration and angiogenesis (Figure 3E).

To determine whether STX17 knockdown affects the migratory behaviour of HUVECs, scratch experiments were performed. ECs migration is crucial in the complex process of angiogenesis.^24^ The results demonstrated that STX17‐knockdown HUVECs, with or without ox‐LDL treatment, significantly inhibited scratch closure at 6 and 12 h compared to the shRNA‐NC group (Figure 3F, G).

Simultaneously, to assess the impact of STX17 knockdown on the angiogenic potential of HUVECs, tube formation assays were conducted. The results showed that STX17‐knockdown HUVECs, irrespective of ox‐LDL treatment, exhibited decreased tube formation in terms of quantity and size compared to the shRNA‐NC group (Figure 3H–J).

These findings collectively suggest that the suppression of STX17 markedly increased HUVECs' susceptibility to ox‐LDL stimulation, diminished their migratory capacity and adversely affected both the quantity and quality of tube formation.

### 3.4 Knockdown of STX17 hinders autophagy flux and impairs lysosomal function

Based on the above results demonstrating the impact of STX17 knockdown on HUVECs function, we explored the mechanisms underlying these impairments. Previous research has emphasized the pivotal role of STX17 in mediating the fusion of autophagosomes and lysosomes, which is crucial for maintaining the stability of autophagy flux.^16^ Consequently, we investigated changes in the expression of autophagy‐related proteins, LC3 and P62, in STX17‐knockdown HUVECs.

**FIGURE 4:**
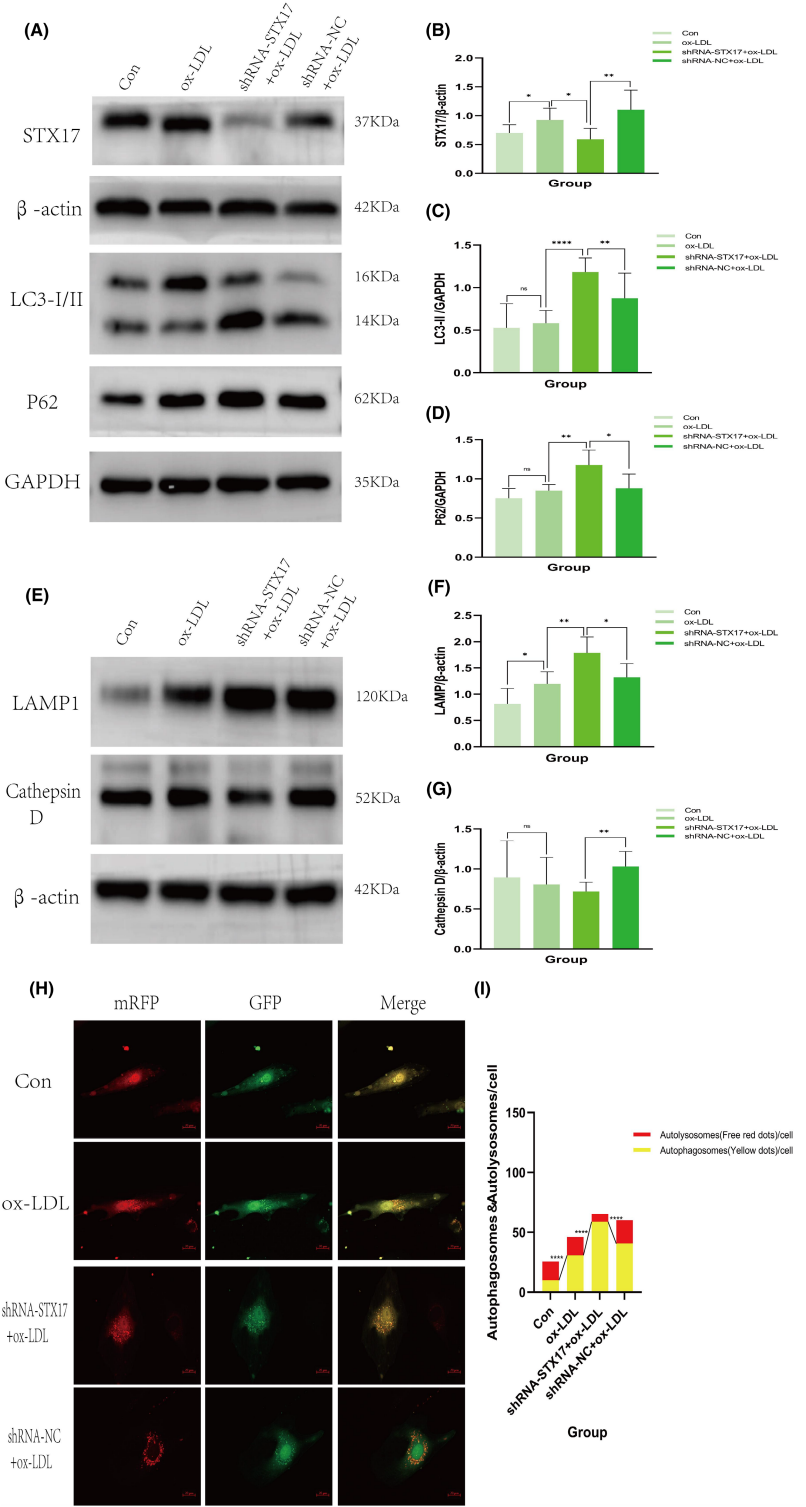
Knockdown of STX17 hinders autophagy flux and impairs lysosomal function in HUVECs treated with ox‐LDL. (A–G) HUVECs, including the control without ox‐LDL treatment, control, shRNA‐STX17 or shRNA‐NC with ox‐LDL treatment (100 μg/mL for 48 h), were harvested and subjected to Western blotting. Representative WB images of STX17 (A and B, *n* = 6), LC3 II (A and C, *n* = 6), P62 (A and D, *n* = 6), LAMP1 (E and F, *n* = 6) and cathepsin D (E and G, *n* = 6) are shown and presented in a bar graph. The bar represents mean ± SD. (H, I) HUVECs infected with shRNA‐STX17 or shRNA‐NC lentivirus and the control HUVECs were transfected with mRFP‐GFP‐LC3 double‐labelled adenovirus for 24 h, then treated with ox‐LDL (100 μg/mL for 48 h). Representative fluorescent micrographs are shown. The mean number of yellow puncta representing autophagosomes and the mean number of red puncta representing autolysosomes are plotted. Scale bar = 20 μm. *p < 0.05, **p < 0.01, ****p < 0.0001 versus control.

We initially confirmed the reduction in STX17 expression in HUVECs transfected with shRNA‐STX17 lentivirus (Figure 4A,B). Notably, without ox‐LDL treatment, there were no significant changes in the expression levels of LC3 and P62 after STX17 knockdown, indicating no alteration in basal autophagy (results not shown). However, under ox‐LDL treatment, the expression of LC3 II (Figure 4A,C) and P62 (Figure 4A,D) significantly increased, suggesting that STX17 knockdown could impede autophagy flux, impair autophagosome degradation, leading to the accumulation of LC3 II and P62.

MRFP‐GFP‐LC3 double‐labelled adenovirus is commonly employed for monitoring autophagy flux, utilizing mRFP and GFP to label and track LC3. The sensitivity of GFP fluorescence to acidity allows the detection of lysosomal fusion with autophagosomes to form autolysosomes, where only mRFP (red) remains detectable. Autophagosomes and autolysosomes were labelled as yellow (mRFP and GFP overlap) and red (mRFP only) dots, and the ratio of yellow to red spots typically expressed autophagy flux. Following STX17 knockdown, the proportion of yellow to red spots (autophagosomes/autolysosomes) significantly increased in ox‐LDL‐treated HUVECs for 48 h (Figure 4H,I).

These results collectively indicate that STX17 knockdown leads to autophagosome accumulation in HUVECs, and impeding autophagy flux.

Conversely, we explored whether STX17 knockdown affects lysosomal function in HUVECs. Subsequently, the expression of lysosomal markers LAMP1 and cathepsin D in STX17‐knockdown HUVECs was investigated. The results revealed no change in the expression levels of LAMP1 and cathepsin D without ox‐LDL treatment (not shown in the figure). However, under ox‐LDL stimulation, increased protein expression of LAMP1 was detected in HUVECs (Figure 4E,F). In contrast, the observed expression levels of cathepsin D (Figure 4E,G) significantly decreased, indicating significant impairment in lysosomal function, which implies that the knockdown of STX17 can result in the accumulation of impaired lysosomes.

### 3.5 Knockdown of STX17 exacerbates ox‐LDL‐induced HUVECs inflammatory response

In addition to autophagy, inflammatory responses play an important role in the context of AS. Afterwards, we further explored whether the knockdown of STX17 affects the inflammatory response of HUVECs.

**FIGURE 5:**
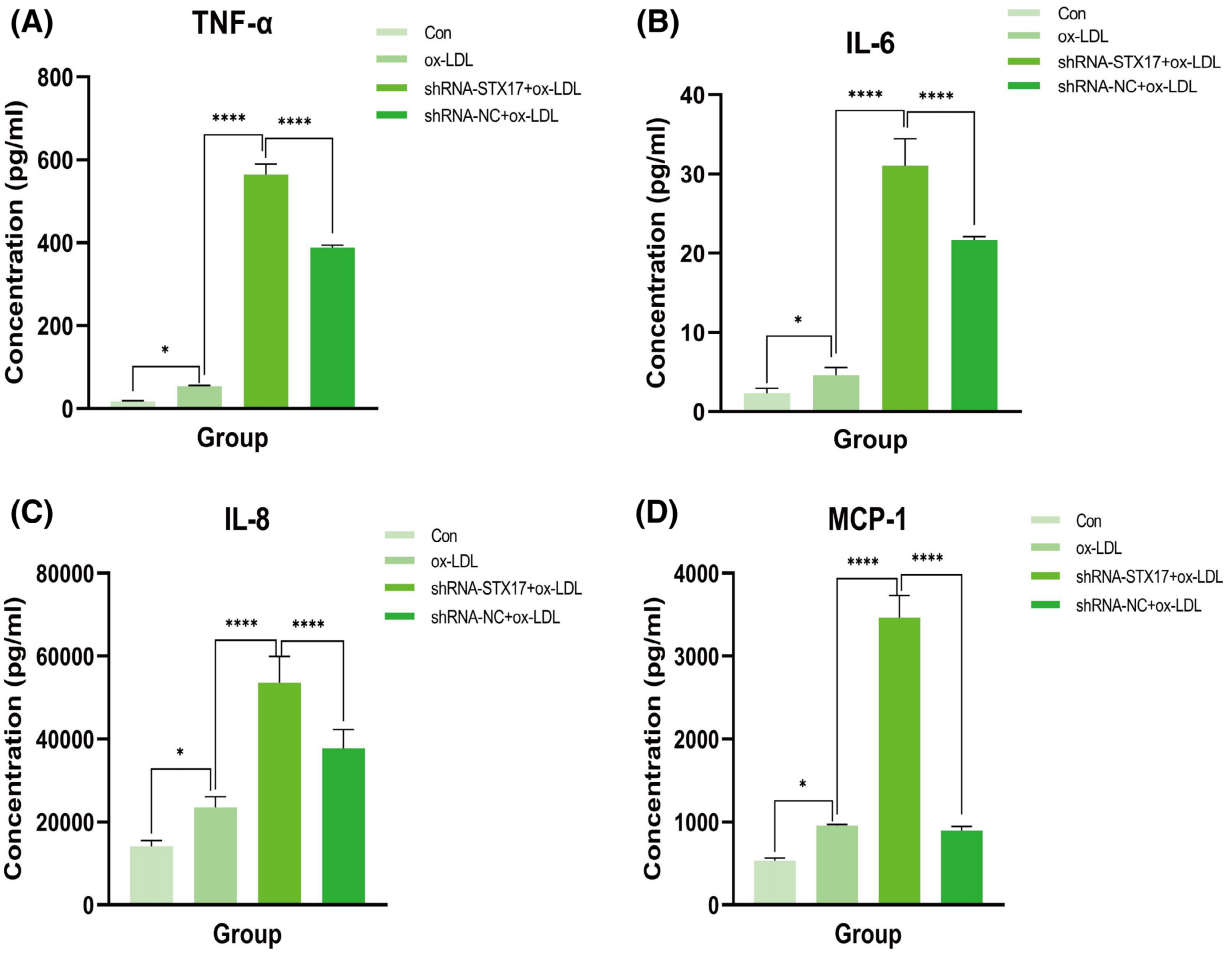
Knockdown of STX17 enhanced ox‐LDL‐induced HUVECs inflammatory response. HUVECs infected with shRNA‐STX17 or shRNA‐NC lentivirus, along with control HUVECs, were exposed to 100 μg/mL ox‐LDL for 48 h. The relative production of TNF‐α (A), IL‐6 (B), IL‐8 (C) and MCP‐1 (D) was detected by ELISA. *p < 0.05, ****p < 0.001 versus control.

To address this, we measured the levels of tumour necrosis factor‐α (TNF‐α), interleukin‐6 (IL‐6), interleukin‐8 (IL‐8) and monocyte chemoattractant protein‐1 (MCP‐1) in the supernatant of HUVECs stimulated by ox‐LDL. The results revealed that compared to the NC group, knockdown of STX17 significantly elevated the levels of TNF‐α (Figure 5A), IL‐6 (Figure 5B), IL‐8 (Figure 5C) and MCP‐1 (Figure 5D) in the supernatant of HUVECs. These findings suggest that STX17 knockdown could exacerbate the inflammatory response of HUVECs, potentially contributing to the progression of AS.

### 3.6 STX17 interacts with STING

**FIGURE 6:**
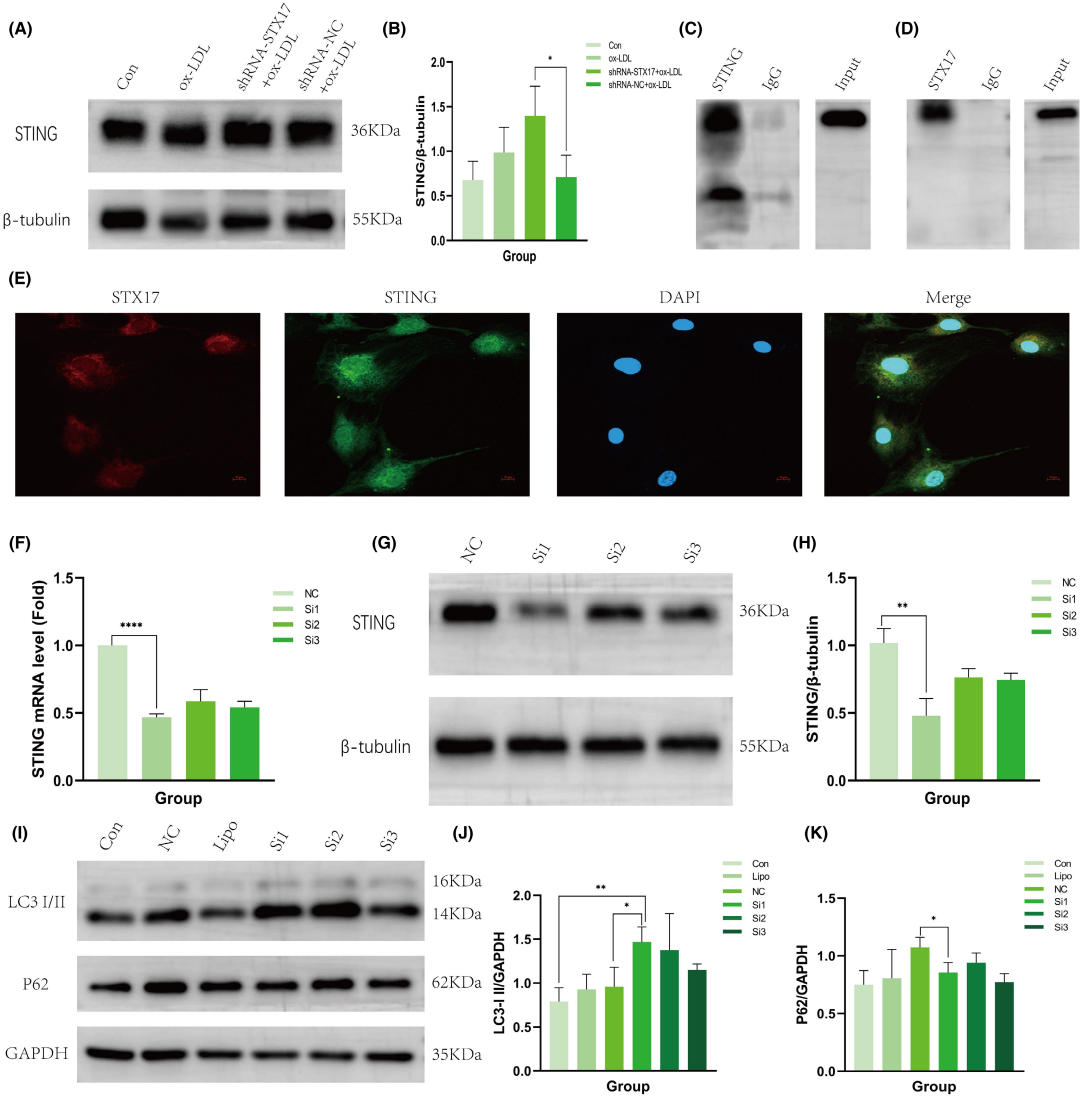
Interaction between STX17 and STING and knockdown of STING promote autophagy flux. (A, B) STING expression in HUVECs infected with shRNA‐STX17 or shRNA‐NC lentivirus and control HUVECs was evaluated through Western blot. Representative WB images are shown and presented in a bar graph (*n* = 3). Bar represents mean ± SD. (C, D) Co‐immunoprecipitation (Co‐IP) was performed in lysates of primary HUVECs. Representative images are shown. (E) Representative fluorescent micrographs are shown. Red: STX17; Green: STING; Blue: DAPI. Scale bar represents 20 μm. (F‐H) At passage 6 of HUVECs, cells were transfected with STING‐siRNA and siRNA‐NC, respectively. The efficiency of STING knockdown was evaluated using PCR (F) and Western blot (G and H). STING expression levels are shown in Western blots (G) and presented in a bar graph (H, *n* = 3). Bar represents mean ± SD. (I–K) Control, STING‐siRNA and siRNA‐NC transfected HUVECs with ox‐LDL treatment (100 μg/mL for 48 h) were harvested and subjected to Western blotting. Representative WB images of LC3 II (I and J, *n* = 3), P62 (I and K, *n* = 3) are shown and presented in a bar graph. Bar represents mean ± SD. *p < 0.05, **p < 0.01, ****p < 0.0001 versus control.

To investigate the potential interaction between STX17 and STING, we initially assessed the expression level of STING in STX17‐knockdown HUVECs. The findings revealed a substantial elevation in the expression level of STING in STX17‐knockdown HUVECs, indicating a correlation between STX17 and STING (Figure 6A,B).

Further evidence for the interaction between STX17 and STING was provided through Co‐IP and immunofluorescence co‐localization. Co‐IP results demonstrated that STING protein was abundant in the anti‐STX17 protein solution but not abundant in the IgG group (Figure 6C). Conversely, STX17 protein was abundant in the anti‐STING protein solution, but not abundant in the IgG group (Figure 6D). In addition, immunofluorescence staining revealed co‐localization of STX17 and STING within the cytoplasm (Figure 6E).

These results collectively provide strong evidence supporting the existence of an interaction between STX17 and STING.

### 3.7 Knockdown of STING ameliorates autophagy flux in HUVECs

Our findings suggest the existence of an interaction between STING and STX17, and previous studies have confirmed that in the absence of stress, STING can physically combine with STX17, which sequesters STX17 within the endoplasmic reticulum. However, this interaction can be disrupted by STING activators or autophagic stimuli, allowing STX17 to translocate to autophagosomes and facilitating membrane fusion and autophagy flux. When the expression of STING increased, the transportation of STX17 from the endoplasmic reticulum to autophagosomes reduced.^19^ This process inhibits the fusion of autophagosomes and lysosomes, consequently impeding autophagy flux. In this study, we further explored the impact of STING knockdown on autophagy flux.

Initially, STING‐siRNA was introduced into HUVECs to induce a reduction in the expression of STING, with scrambled siRNA(siRNA‐NC) as a comparative control. PCR (Figure 6F) and WB (Figure 6G,H) were utilized to validate the expression level of STING in HUVECs. The results demonstrated that the expression level of STING in HUVECs transfected with STING‐siRNA was approximately 50% of that observed in HUVECs transfected with siRNA‐NC.

Subsequently, we elucidated the expression levels of LC3 and P62 in STING‐knockdown HUVECs. The findings revealed a significant upregulation in the expression of LC3 II (Figure 6I,J) in STING‐knockdown HUVECs upon stimulation with ox‐LDL, while the expression of P62 was notably diminished (Figure 6I,K). These results indicate that autophagy was induced and autophagy flux was activated. Consequently, these findings substantiate that STING knockdown could nullify the influence of STX17 on autophagy flux.

## 4 DISCUSSION

Autophagy, an intracellular degradation system dependent on lysosomes, has recently become a research hotspot. Through this system, substances such as ageing organelles, abnormal proteins and invading pathogens are degraded into small molecules within lysosomes, thereby supplying essential nutrients and materials for cellular self‐renewal.^25^ Research has established a close association between autophagy and AS.^26^ Notably, the AS microenvironment contains numerous autophagic stimuli, including reactive oxygen species (ROS), inflammatory mediators, low‐density lipoprotein cholesterol (LDL‐C) and TNF‐α.^27^ Autophagy in the context of AS exhibits a dualistic impact. When maintained at a moderate or controlled level, autophagy serves as a safeguard for vascular cells by facilitating the degradation of impaired organelles and proteins via autolysosomes. This process effectively diminishes the area of arterial plaques, curtails lipid deposition and inflammatory cell aggregation, fosters the transformation of macrophages into the anti‐inflammatory M2 subtype and mitigates cell demise within the plaques.^28^ Conversely, excessive activation of autophagy exacerbates the progression of AS. Severe oxidative stress or inflammation can result in an excessive induction of autophagy, leading to autophagy‐dependent cell death, thinning of fiber caps, destabilization of plaques, thrombosis, restenosis and acute coronary events.^29^ Autophagy flux is a continuous and dynamic process, strictly regulated by autophagy‐related genes.^30^ STX17, a member of the SNAREs family, participates in the fusion process of autophagosomes and lysosomes. It is now understood that STX17 on autophagosomes binds to SNAP29 and VAMP8 on lysosomes, driving the fusion of autophagosomes with lysosomes.^31^ Research has demonstrated that the inhibition of autophagy flux plays an important role in cardiovascular diseases, especially AS, which is the fundamental pathological mechanism underlying CVDs.^32^ The obstruction of autophagy flux is more prevalent in AS, which may be a crucial determinant of AS.

In this study, an in vivo AS model was constructed by feeding ApoE KO mice a high‐fat diet for 12 weeks, and an in vitro AS model was established by stimulating HUVECs with ox‐LDL at a concentration of 100 μg/mL for 48 h. Both models exhibited upregulation of STX17 expression. The reason for the increased expression of STX17 consists of two parts. Firstly, in the context of AS, autophagy is overactivated, which means that the production of autophagosomes increases, leading to the increased expression of STX17, which exists on autophagosomes. Secondly, obstruction of autophagy flux is more ordinary in AS, which may result in reduced degradation of autophagosomes. Both the reasons can increase the expression of STX17. Subsequently, we knocked down the expression of STX17 in the in vitro AS model, revealing that the knockdown of STX17 had detrimental effects on ox‐LDL‐stimulated HUVECs.

Importantly, we investigated whether knockdown of STX17 has a potential involvement in ox‐LDL‐induced HUVECs injury. We explored the effects of STX17‐knockdown HUVECs from several aspects such as endothelial cell proliferation, migration and angiogenesis. The proliferation of HUVECs was assessed using the CCK8 assay, and it was revealed that transfection with shRNA‐STX17 or shRNA‐NC lentivirus did not affect their growth rate. Experiments were conducted to exclude the influence of different cell proliferation trends on subsequent migration and angiogenesis experiments. It is well‐established that HUVECs migration is necessary for endothelial cell angiogenesis.^33^ Accordingly, we initially investigated the impact of STX17 knockdown on the migration ability of HUVECs. The results displayed that STX17‐knockdown HUVECs significantly inhibited the closure of scratches at 6 h and 12 h with or without ox‐LDL treatment. Additionally, tube‐formation experiments further revealed that STX17‐knockdown HUVECs produced fewer and smaller tubes, regardless of ox‐LDL treatment.

We then further explored the mechanism causing damage to HUVECs after knocking down STX17. As mentioned in previous research, STX17 participates in the fusion of autophagosomes and lysosomes. We then considered the autophagic effect of STX17 knockdown. In STX17‐knockdown HUVECs without ox‐LDL treatment, there were no significant alterations observed in the expression levels of LC3 and P62, indicating that the knockdown of STX17 did not impact the basal autophagy of HUVECs. This because through lentivirus transfection, we only knockdown about 50% of STX17 expression in HUVECs. Without ox‐LDL stimulation, autophagy in HUVECs remained at baseline level. Although the expression of LC3 and P62 slightly increased, the *p* value has no statistical significance. Nevertheless, upon ox‐LDL treatment, the expression levels of LC3 II and P62 in STX17‐knockdown HUVECs exhibited a significant increment. During the initiation of autophagy, the enzymatic hydrolysis of cytoplasmic LC3 I results in the removal of a small peptide segment, which then binds to phosphatidylethanolamine (PE) and transforms into membrane‐type LC3 II. Therefore, the expression level of LC3 II can be estimated as an indicator of the activation level of autophagy.^34^ During the process of autophagosome formation, P62 is selectively encapsulated within the autophagosome and subsequently degraded by proteolytic enzymes in the autolysosome. As a result, the expression level of P62 exhibits a negative correlation with autophagic activity.^35^ The findings of this experiment indicate that with ox‐LDL treatment, the formation of autophagosomes and the expression of LC3 II were elevated in STX17‐knockdown HUVECs, but the fusion of autophagosomes and lysosomes was impaired, P62 degradation reduced, which means blockage of autophagy flux. The utilization of mRFP‐GFP‐LC3 double‐labelled adenovirus is a classic technique for observing autophagy flux. The findings of this experiment displayed that the yellow ratio in STX17‐knockdown HUVECs exhibited a significant increase than that of red with ox‐LDL treatment, providing additional evidence that the fusion of autophagosomes and lysosomes is blocked, hindering autophagy flux.

This study additionally discovered that the knockdown of STX17 could lead to lysosomal dysfunction in HUVECs with ox‐LDL treatment. WB analysis displayed that the expression of the lysosomal marker LAMP1 was notably increased in STX17‐knockdown HUVECs, while the expression of the lysosomal protease cathepsin D was downregulated, aligning with prior research findings. [Gly14]‐humanin (HNG), a derivative of HN, is a mitochondrial peptide widely present in animals, with neuroprotective and cytoprotective properties. Research has revealed that HNG can effectively enhance lysosomal function and autophagy of HUVECs treated with ox‐LDL, mitigating the decreased expression of cathepsin D induced by ox‐LDL treatment.^36^ Coxsackievirus B3 (CVB3) is an important human pathogen linked to arrhythmia and acute heart failure. Studies have demonstrated that CVB3 infection could diminish the expression of STX17 in HeLa cells, which not only impedes autophagy flux but also induces dysfunction of lysosomes, ultimately resulting in a decline in the expression of cathepsin B/L.^37^ Based on the aforementioned findings, the downregulation of STX17 in HUVECs resulted in an upregulation of LAMP1 expression, implying an augmentation in lysosomal quantity. However, the reduced expression of cathepsin D indicated impaired lysosomal functionality. Consequently, it can be inferred that the knockdown of STX17 not only impedes the fusion of autophagosomes and lysosomes, obstructing autophagy flux but also induces lysosomal dysfunction.

Previous studies have demonstrated that inflammation plays a pivotal role in all phases of atherosclerotic plaque development, from the initiation of fatty streaks to the eventual rupture of the plaque.^38^ Therefore, directing therapeutic interventions toward inflammatory pathways holds significant potential for the prevention and management of AS. A notable illustration of this is the CANTOS study, which revealed that, based on lipid‐lowering treatment, the incidence of adverse cardiovascular events in patients with AS could be further reduced about 15% by giving the anti‐IL‐1β antibody Canakinumab.^39^ We then assessed whether STX17 knockdown affects the inflammatory response of HUVECs in the context of AS. ECs have the capability to release many pro‐inflammatory mediators, including TNF‐α, IL‐6, IL‐8 and MCP‐1, facilitating the onset and progression of AS.^23^ The expression of these mediators further facilitates the recruitment of macrophages, the differentiation of monocytes and the deposition of oxidized lipids, ultimately leading to the formation of foam cells and lipid pools.^40^ In this study, to investigate the effect of knockdown of STX17 on the inflammatory response of ox‐LDL‐induced HUVECs, we measured the levels of TNF‐α, IL‐6, IL‐8 and MCP‐1 in the HUVECs supernatant. The results showed that compared to the shRNA‐NC group, the concentration of TNF‐α in STX17‐knockdown HUVECs increased from 53.55 pg/mL to 564.43 pg/mL, about a 10‐fold increase; The concentration of IL‐6 increased from 2287.47 pg/mL to 15523.82 pg/mL, about a 7‐fold increase; The concentration of IL‐8 increased from 23471.51 pg/mL to 53541.12 pg/mL, about a 2‐fold increase; Finally, the concentration of MCP‐1 increased from 955.31 pg/mL to 3464.56 pg/mL, about a 4‐fold increase. The above results all suggest that knockdown of STX17 in HUVECs exacerbates the inflammatory response with ox‐LDL treatment. Therefore, we speculate that knockdown of STX17 not only exacerbates AS by blocking autophagy flux but also promotes the occurrence and development of AS through intensified inflammatory response.

As mentioned in previous research, STX17 is primarily implicated in the fusion of autophagosomes and lysosomes. However, this study reveals that knockdown of STX17 exacerbates the inflammatory response in HUVECs, prompting further exploration into the potential mechanisms. STING, serving as a key adaptor protein in the innate immune system, plays a crucial role in the host defence against external pathogen invasions. Simultaneously, STING exhibits characteristics akin to inflammatory molecules and a close association with the inflammatory response.^41^ Elevated or overactivated expression of STING or its related signalling pathway can lead to severe infectious diseases, non‐infectious diseases and autoimmune conditions due to excessive inflammatory reactions.^42^ Recent studies have provided evidence that STING mediates pro‐inflammatory activation of macrophages during the occurrence and progression of AS,^43^ and can induce endothelial‐to‐mesenchymal transition (EndMT) in ECs, accelerating endothelial dysfunction.^44^ Recent evidence suggests that STING can physically interact with STX17.^19^ Therefore, in this study, we considered whether STING could interact with STX17 to participate in autophagy in the context of AS. Firstly, we explored the interaction between STING and STX17 using Co‐IP and immunofluorescence staining techniques. The findings from Co‐IP and immunofluorescence staining confirmed the interaction between STING and STX17. Additionally, the expression level of STING was significantly higher in STX17‐knockdown HUVECs than in other groups, providing further evidence for the interaction between STING and STX17. Secondly, we aimed to clarify the involvement of STING in the autophagy of HUVECs in AS. To achieve this, we employed STING‐siRNA and siRNA‐NC to knock down the expression level of STING in HUVECs. The efficiency of STING knockdown was confirmed through PCR and WB analysis. Subsequently, autophagy activity was observed in ox‐LDL‐treated HUVECs after STING knockdown. The results demonstrated a significant increase in the expression level of LC3 II, while the expression level of P62 significantly decreased, indicating that autophagy was induced and autophagy flux was activated. According to the aforementioned findings and previous studies, it can be postulated that inhibiting the expression of STING in vivo can increase the transport of STX17 from the endoplasmic reticulum to autophagosomes, facilitate the fusion of autophagosomes and lysosomes, augment the stability of autophagy flux, and consequently ameliorate the advancement of AS. In the future, we will further explore the ramifications of overexpression of STX17 on HUVECs in the context of AS.

There are several limitations in this study. First, most of experiments were conducted in vitro, therefore, further exploration is needed to determine whether the same results can be obtained in EC‐specific STX17 knockout mice. Second, the mechanism by which STX17 exacerbates AS has not been fully explored. In addition to autophagy and inflammation, STX17 may also play a certain role in other aspects, such as apoptosis. Third, STX17 only a small part of a larger complex of proteins required for efficient and regulated autophagosome‐lysosome fusion. Whether other proteins, for example, ATG14 (an essential autophagy‐specific regulator of the class III phosphatidylinositol 3‐kinase complex, also participates in the fusion process of autophagosomes and lysosomes), involved in the autophagy process of AS needed further exploration. Fourth, hyperlipidemia plays an important role in the occurrence and development of AS, and further investigation is warranted to explore whether STX17 knockdown regulates AS by affecting blood lipid levels.

In summary, our research findings revealed that the expression level of STX17 was upregulated in both in vivo and in vitro AS models. Knockdown of STX17 exacerbates the function of HUVECs, and results in blockage of autophagy flux and accumulation of dysfunctional lysosomes. Furthermore, knockdown of STX17 aggravates the inflammatory response of ox‐LDL‐treated HUVECs, further worsening the condition of AS. Simultaneously, STX17 interacts with STING. Knockdown of STX17 increases STING expression, while knockdown of STING improves the autophagy flux of ox‐LDL‐treated HUVECs, suggesting that STING may affect autophagy flux in AS by interacting with STX17.

## AUTHOR CONTRIBUTIONS

**Xinyue Cui:** Writing – original draft (lead). **Bo Wang:** Writing – review and editing (lead). **Dongjian Han:** Conceptualization (lead). **Mengdie Cheng:** Methodology (lead). **Peiyu Yuan:** Data curation (lead). **Pengchong Du:** Software (lead). **Yachen Hou:** Formal analysis (lead). **Chang Su:** Resources (lead). **Junnan Tang:** Project administration (lead). **Jinying Zhang:** Project administration (supporting); supervision (lead).

## FUNDING INFORMATION

This work was supported by the National Natural Science Foundation of China (No. U2004203), the Special Support for High Level Talents in Henan Province (No. ZYQR201912131), and Henan Provincial Medical Science and Technology Tackling Program Project (LHGJ20210334 and SBGJ202101012).

## CONFLICT OF INTEREST STATEMENT

The authors declare that they have no known competing financial interests or personal relationships that could have appeared to influence the work reported in this paper.

## Data Availability

The data that support the findings of this study are available from the corresponding author upon reasonable request.
